# Comparison of Supraclavicular Regional Nerve Block Versus Infraclavicular Regional Nerve Block in Distal Radial Open Reduction and Internal Fixation: A Retrospective Case Series

**DOI:** 10.7759/cureus.24079

**Published:** 2022-04-12

**Authors:** Promil Kukreja, Alexander M Kofskey, Erin Ransom, Chelsea McKenzie, Joel Feinstein, Jared Hudson, Hari Kalagara

**Affiliations:** 1 Anesthesiology and Perioperative Medicine, University of Alabama at Birmingham, Birmingham, USA; 2 Orthopedic Surgery, University of Alabama at Birmingham, Birmingham, USA; 3 Department of Anesthesiology and Perioperative Medicine, Mayo Clinic, Jacksonville, USA

**Keywords:** postoperative analgesia, orif distal radius, oral morphine equivalents, infraclavicular block, supraclavicular block

## Abstract

Background

The management of pain in patients undergoing open reduction and internal fixation (ORIF) of distal radius fractures (DRFs) remains an area of debate for anesthesiologists due to a variety of block options and no definitive superior technique among these modalities. In this retrospective case series, we compare the efficacy of supraclavicular versus infraclavicular regional nerve blocks for surgical patients undergoing distal radial ORIF operations to determine if one approach was superior.

Methodology

This retrospective case series included patients undergoing ORIF of a DRF at a tertiary academic medical center between April 28, 2016, and August 23, 2021. In total, 54 patients undergoing ORIF of a DRF provided written consent for the nerve block(s) on the day of surgery. Of these 54 patients, 54 (100%) underwent primary procedures. Of the 54 primary ORIF patients, 28 (52%) received the supraclavicular block, while 26 (48%) received the infraclavicular nerve block.

Results

The infraclavicular and supraclavicular groups did not significantly differ regarding age, gender, American Society of Anesthesiologists, weight, or body mass index. The primary (intraoperative opioid use) and secondary (postoperative opioid use, postoperative nausea and vomiting in the post-anesthetic care unit, highest and average pain scores, and time to discharge) outcomes data were included in the study. The infraclavicular and supraclavicular groups did not significantly differ in any of the assessed outcomes except for time to discharge.

Conclusions

The supraclavicular block approach for distal radius ORIF offers an effective and non-inferior alternative to the infraclavicular block approach concerning effective analgesia and safety.

## Introduction

Distal radial fractures are frequently encountered in both the emergency department and operating room settings, accounting for 8-15% of all bony injuries in adults [1]. Similar to the rest of the arm, the distal radius is innervated by the brachial plexus. The anterior forearm sensation is supplied by cervical roots 6-8 while the posterior forearm sensation is supplied by cervical roots 5-8. To provide adequate analgesia to the wrist, coverage in the median, ulnar, and radial nerve distribution is required [2]. Thus, there are different sites of brachial plexus blocks that may be utilized to cover the nerve distribution of the distal radius. The variety of block options has led to much discussion and investigation as to which regional anesthetic technique may be the most optimal for postoperative analgesia following distal radial surgery. Traditionally, supraclavicular blocks are utilized for surgeries of the upper extremity distal to the shoulder, infraclavicular blocks are utilized for surgeries distal to the axilla, and axillary blocks are utilized for surgeries at or distal to the elbow [3]. This study examines and compares the utility of supraclavicular and infraclavicular regional nerve blocks for surgical patients undergoing distal radial operative reduction and fixation (ORIF) operations.

Although the supraclavicular brachial plexus block approach can provide effective anesthesia for upper extremity surgery, its routine use for distal radius ORIF or hand surgery is not well established. When compared with axillary or infraclavicular brachial plexus block, supraclavicular block offers a distinct advantage of fast and dense block [4]. As supraclavicular brachial plexus anatomy has pleura and subclavian artery in close vicinity, different approaches have been described to minimize the risks of pneumothorax and vascular puncture [5,6]. The use of ultrasound guidance for the supraclavicular block has significantly improved the safety margin and the quality of the block and has shortened the time taken to perform the block [7]. Soares et al. have described the “corner pocket” supraclavicular block approach for optimal needle tip position for local anesthetic administration [8]. The infraclavicular block has been routinely used as a safe and effective regional block for distal radius and hand surgery [3].

Block onset time and effective surgical anesthesia are clinically important for minor wrist and hand surgery. There are only a few prospective studies comparing the “corner pocket” supraclavicular block with infraclavicular block when administered as surgical anesthesia for wrist and hand surgery [9]. In our retrospective study, we compared these two blocks for postoperative analgesia as a primary outcome.

## Materials and methods

This retrospective case series included patients undergoing ORIF of a distal radius fracture (DRF) at a tertiary academic medical center between April 28, 2016, and August 23, 2021. The Institutional Review Board (IRB) of the University of Alabama at Birmingham issued approval (IRB project number: 300000976). In total, 54 patients undergoing ORIF of a DRF provided written consent for the nerve block(s) on the day of surgery. Of the 54 patients, 54 (100%) underwent primary procedures. Of the 54 primary ORIF patients, 28 (52%) received the supraclavicular block, while 26 (48%) received the infraclavicular nerve block. All nerve blocks were performed preoperatively with patients awake and in a supine position in a dedicated “block” area. The blocks were performed by a regional anesthesia fellow or senior resident under the direct supervision of an experienced regional anesthesia faculty. The ORIF was performed under general anesthesia based on a routine. The visual analog scores of pain were obtained every 15 minutes in the post-anesthetic care unit (PACU) after surgery. In addition, usage of postoperative nausea and vomiting (PONV) medication was recorded as either used or not used.

Data were summarized using means and standard errors (SE) for continuous outcomes or counts and percentages for categorical outcomes. Two-sample t-tests and chi-square tests were used to compare the two groups. Normality for continuous outcomes was assessed using probability plots and the Shapiro-Wilk test for normality. For any outcomes where normality could not be reasonably assumed, the Wilcoxon rank-sum test was used instead of the t-test. For categorical outcomes, Fisher’s exact test was used when assumptions for the chi-square test were not met. A p-value of <0.05 was considered statistically significant. SAS version 9.4 (SAS Institute Inc., Cary, NC) was used to conduct all statistical analyses.

Ultrasound-guided technique for supraclavicular and infraclavicular blocks

**Figure 1:**
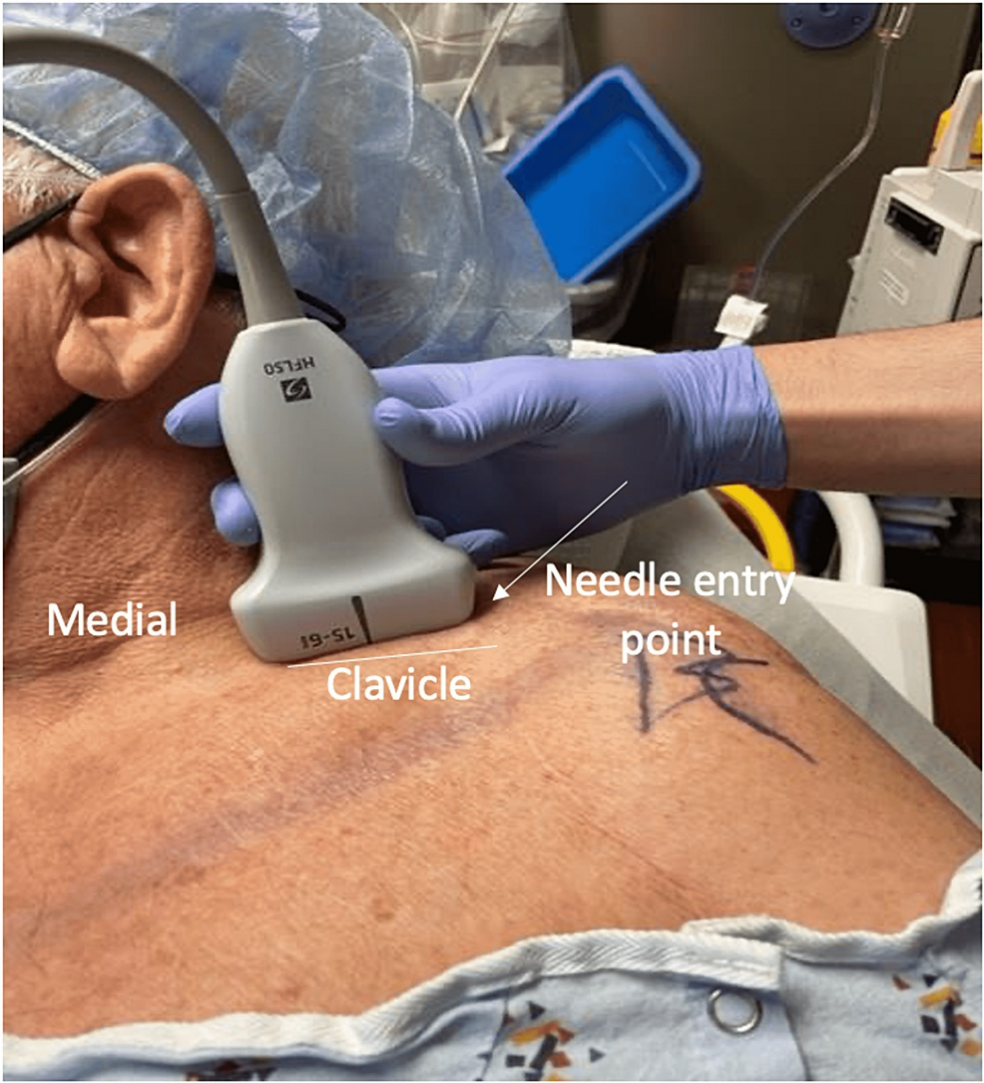
Patient positioned supine with the head turned away from the block side. The linear ultrasound probe placed over the supraclavicular region for the in-plane needle corner-pocket supraclavicular block technique.

**Figure 2:**
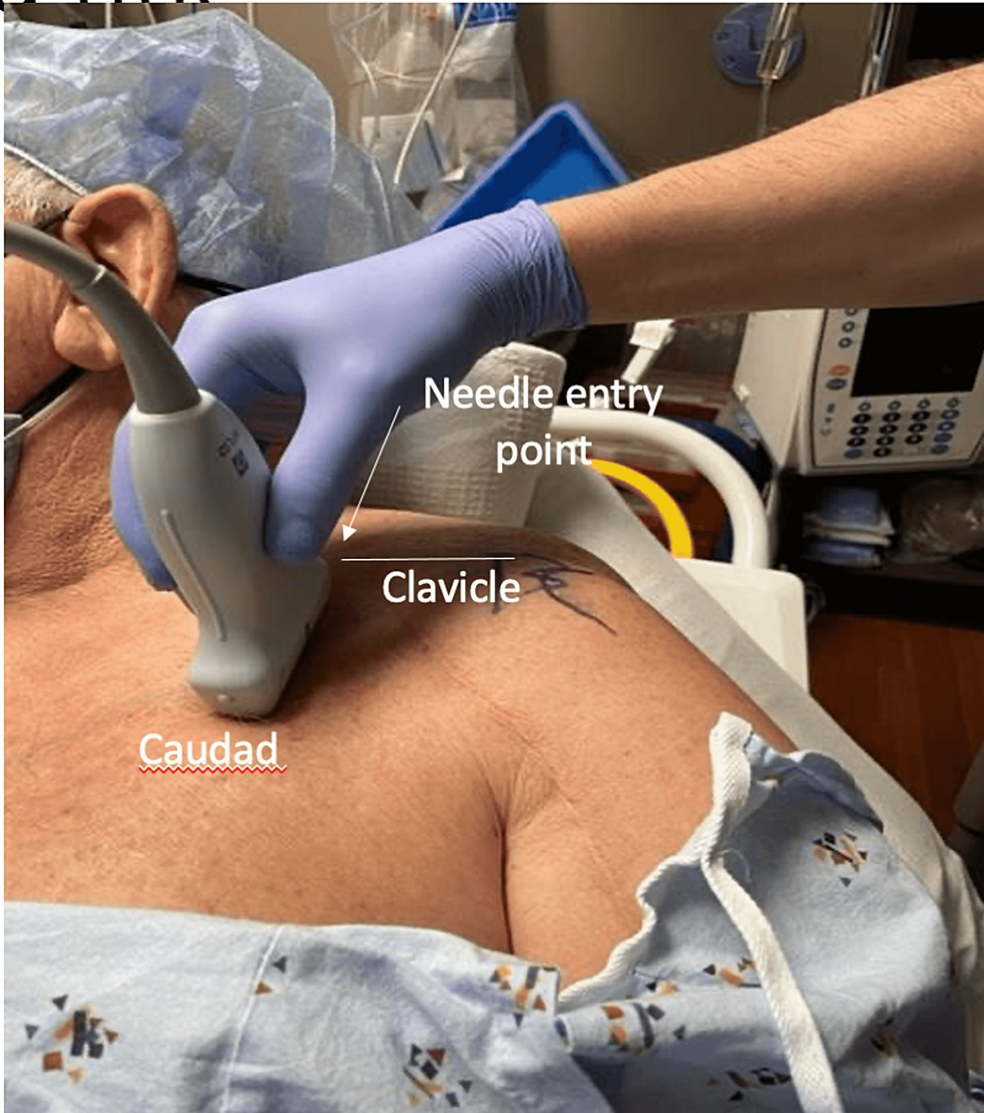
Patient positioned supine with the head turned away from the block side. The linear ultrasound probe placed under the clavicle in the deltopectoral groove for the in-plane needle infraclavicular technique.

**Figure 3:**
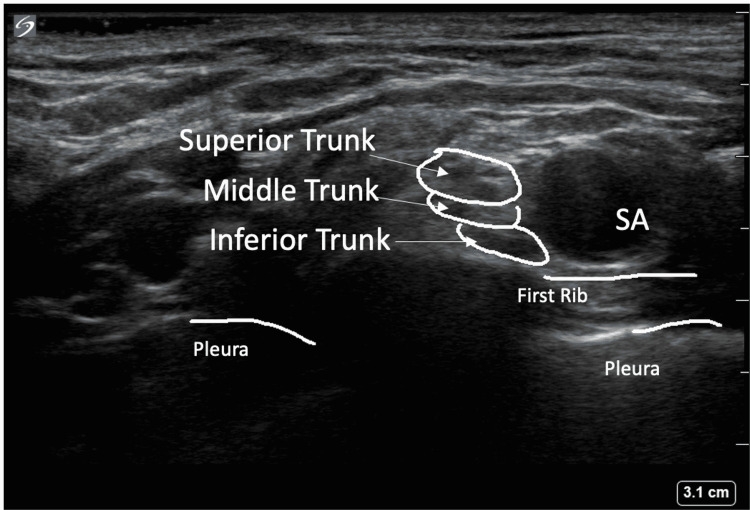
Ultrasound image of brachial plexus trunks at the supraclavicular level in relation to the subclavian artery, first rib, and pleura. SA: subclavian artery

**Figure 4:**
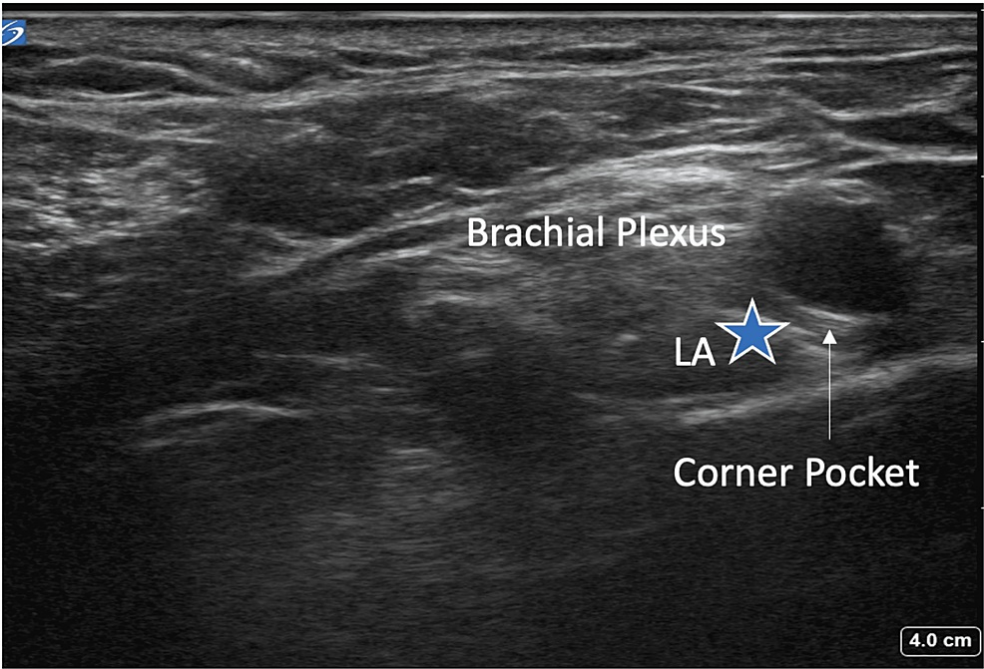
Ultrasound image of the local anesthetic deposited in the corner pocket for the supraclavicular block. LA: local anesthetic

**Figure 5:**
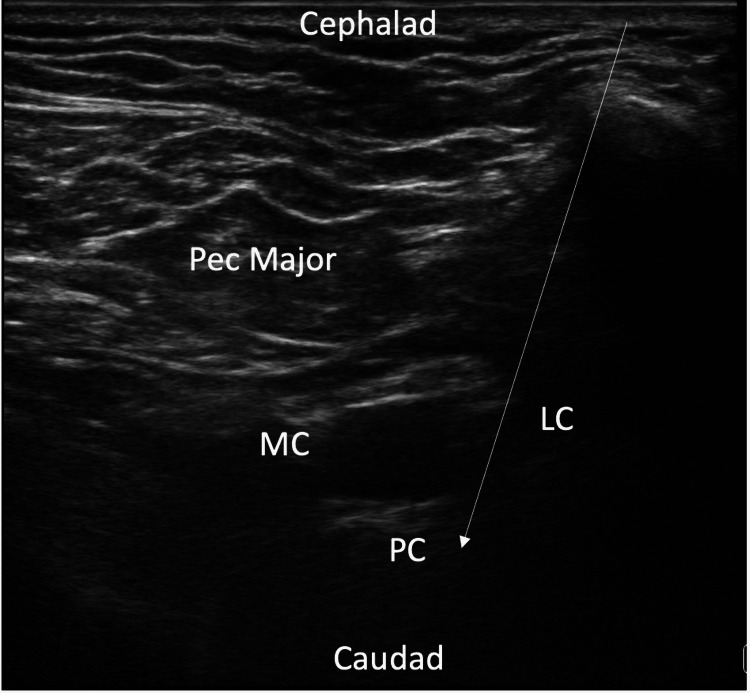
Ultrasound image of brachial plexus cords at the infraclavicular level and needle trajectory for the in-plane block technique. MC: medial cord; LC: lateral cord; PC: posterior cord

The patient was positioned in the supine position (Figure 1), and a high-frequency linear transducer was used to target the trunks of the brachial plexus for the supraclavicular nerve block (corner pocket approach) and the cords of the brachial plexus for the infraclavicular block (Figure 2). For the supraclavicular nerve block, a linear transducer was placed along the supraclavicular fossa to visualize the brachial plexus and the subclavian artery (Figure 3). Using an in-plane technique, a 10-cm, echogenic, 21-gauge needle was advanced lateral to the medial, targeting the brachial plexus with an injection of 25 mL of 0.5% ropivacaine (Figure 4). For the infraclavicular nerve block, the ultrasound probe was placed below the clavicle in the deltopectoral groove (coracoid approach) in a vertical orientation to identify cords in relation to the subclavian artery. A 10-cm, echogenic, 21-gauge needle was advanced to the posterior cord (Figure 5) at the 6 o’clock position, and 25 mL of 0.5% ropivacaine was injected, covering the cords of the brachial plexus.

## Results

Data were available for 54 subjects: 26 with infraclavicular nerve block, 28 with supraclavicular nerve block, and two with axillary nerve block. The two subjects with axillary nerve blocks were excluded from further statistical analysis, leaving a final sample size of 54 subjects.

**Figure 6:**
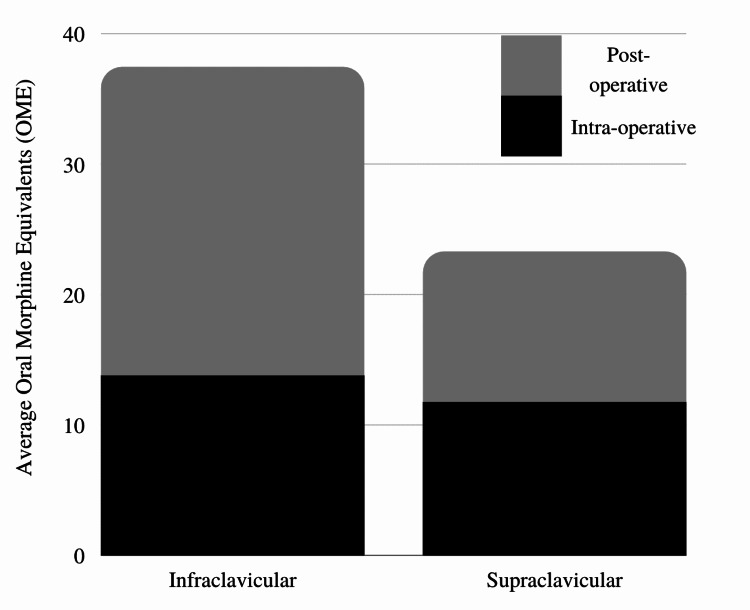
Average oral morphine equivalent usage intraoperatively and postoperatively by group.

Table 1 shows the demographic and clinical characteristics of the sample. The infraclavicular and supraclavicular groups did not significantly differ on age, gender, American Society of Anesthesiologists (ASA), weight, or body mass index (BMI). Table 2 shows the primary (intraoperative opioid use) and secondary (postoperative opioid use, PONV in the PACU, highest and average pain scores, and time to discharge) outcomes of the study. In this study, fentanyl was used as intra and postoperative opioid for pain management. The opioid requirement was documented as oral morphine equivalents (OME) in milligram (mg) unit. Figure 6 shows the differences in average intra and postoperative opioid use for the supraclavicular and infraclavicular groups. The infraclavicular and supraclavicular groups did not significantly differ on any of the assessed outcomes.

**Table 1:** Demographic and clinical characteristics by group. *P-values from Wilcoxon rank-sum test (age), chi-square test (gender, ASA status), or two-sample t-test (weight, BMI). SE: standard error; ASA: American Society of Anesthesiologists; BMI: body mass index

Variable	Infraclavicular (n = 26)	Supraclavicular (n = 28)	P-value*
Age (years), mean (SE)	50.42 (3.31)	52.04 (3.46)	0.768
Gender, N (%)			0.599
Female	13 (50.00%)	16 (57.14%)	
Male	13 (50.00%)	12 (42.86%)	
ASA status, N (%)			0.505
1	3 (11.54%)	2 (7.14%)	
2	12 (46.15%)	11 (39.29%)	
3	11 (42.31%)	13 (46.43%)	
4	0 (0.00%)	2 (7.14%)	
Weight (kg), mean (SE)	77.53 (3.65)	80.29 (3.54)	0.589
BMI (kg/m^2^), mean (SE)	28.07 (1.20)	28.13 (1.00)	0.966

**Table 2:** Primary and secondary outcomes by group. *P-values from Wilcoxon rank-sum test (opioids, pain scores, time to discharge) or chi-square test (PONV). SE: standard error; PACU: post-anesthetic care unit; PONV: postoperative nausea and vomiting; OME: Oral morphine equivalent (mg)

Variable	Infraclavicular (n = 26)	Supraclavicular (n = 28)	P-value*
Opioids (OME), mean (SE)			
Intraoperative	13.77 (1.72)	11.73 (2.07)	0.240
Postoperative	23.64 (8.78)	11.53 (3.81)	0.553
Pain scores in PACU, mean (SE)			
Highest score	2.73 (0.72)	1.92 (0.64)	0.326
Average score	1.77 (0.48)	1.02 (0.32)	0.208
PONV in PACU, N (%)	2 (7.69%)	3 (10.71%)	0.702
Time to discharge (hours), mean (SE)	16.52 (5.97)	7.33 (2.73)	0.024

## Discussion

In this retrospective study, there was no difference between the two regional anesthesia blocks used in terms of intra and postoperative opioid use, postoperative pain scores, and incidence of PONV.

The distal radius fracture is a common orthopedic procedure performed either under general anesthesia or regional anesthesia. It can sometimes result in severe postoperative pain which may interfere with rehabilitation, delayed recovery, and discharge [10]. At our institute, we perform brachial plexus block prior to surgery for postoperative analgesia. In this retrospective study, we compared two different brachial plexus regional anesthesia techniques used for DRF surgery, supraclavicular and infraclavicular nerve blocks.

Infraclavicular blocks are commonly performed as the regional anesthesia technique of choice for open reduction and fixation of DRF [11]. Infraclavicular block targets the brachial plexus at the level of cords and is considered to provide an effective blockade of the radial nerve (posterior cord). The supraclavicular block may spare blocking of the inferior trunk and posterior division which may result in incomplete coverage for DRF surgery. In this study, we used the “corner pocket” approach for the supraclavicular block to ensure adequate coverage of the inferior trunk. Both types of blocks were performed under ultrasound guidance which is established as a safe, effective, and cost-effective method of anesthesia [12,13]. The needle visualization in the supraclavicular approach was superior when compared to the infraclavicular approach even in the high BMI patient population [14]. We did not report the quality of needle visualization after the procedure in our documentation.

Regional anesthetic nerve blocks have demonstrated improved outcomes in terms of postoperative analgesia and hospital discharge speed [15,16]. In this retrospective study, all included patients were discharged the same day after surgery. Both blocks were found to be effective with respect to pain control and opioid consumption. There was no statistical difference in these categories, although pain scores and opioid consumption trended higher in the infraclavicular block group. The “corner pocket” supraclavicular block resulted in more consistent outcomes with respect to pain control and OMEs (Table 2).

Opioid consumption in the PACU is critical for timely discharge to phase II recovery. More opioid consumption may lead to PONV and delay discharge from the hospital for same-day surgery. A recent randomized controlled trial has shown that morphine consumption in the PACU was significantly lower in the regional anesthesia (infraclavicular block) group when compared to the general anesthesia group after DRF fixation [11]. In our retrospective study, both regional anesthesia techniques infra and supraclavicular blocks were compared and there was no statistical difference between opioid consumption during the intra and postoperative period.

Postoperative pain after DRF fixation surgery is not typically an issue and is usually well controlled with regular oral opioids (hydrocodone) in combination with acetaminophen for same-day surgery patients. There is a concern about rebound pain after wearing off the regional block, but a regular oral pain regimen has been shown to attenuate rebound pain [17]. The infraclavicular block itself has shown to have a preventive and prolonged analgesic effect lasting much more than the five times the half-life of the local anesthetic drug used [18]. In our retrospective study, we did not have data for long-term follow-up. Our study was focused on perioperative pain control and hospital stay duration for same-day DRF surgery.

The “corner pocket” supraclavicular block approach may present with a potential risk of pneumothorax due to the proximity of the needle tip and pleura [19]. In our study, there were no serious complications, and, in particular, no pneumothoraxes with the “corner pocket” approach. We did not have data for “block procedure” duration and did not assess the speed of onset for both blocks. A prospective randomized study compared the onset time and did not find any difference in onset time between “corner pocket” supraclavicular block versus infraclavicular block [9].

Wong et al. have concluded that patient satisfaction was significantly higher in the patient population who received regional anesthesia in the early postoperative period [11]. Myles et al. also demonstrated that patient dissatisfaction is strongly associated with worse pain control as well as PONV [20]. In our study, we did not report patient satisfaction, but the incidence of PONV was similar in both groups. Although hospital stay duration was significantly less in the supraclavicular block group. The shorter stay in this group may be attributed to less opioid consumption, better pain control, or less PONV, which were not significantly different from the infraclavicular group.

There were some limitations in this study. It was a retrospective study with a small sample size and was conducted at a single center. For rare outcomes such as the incidence of pneumothorax, a large sample size is needed. There is a risk of recall and selection bias with retrospective studies. The retrospective nature of the study limited the ability to collect data beyond hospital discharge for short-term and long-term pain scores, opioid consumption, and functional outcomes. Different medications delivered for the block and during the operation also present a limitation for this study. Moreover, there was no control group to compare the benefits of both types of regional anesthesia over general anesthesia. Some relevant data points such as block procedure time, ease of needle visualization, and block onset times were not documented.

## Conclusions

In the context of a clinical setting where each approach of brachial plexus block possesses pros and cons, the supraclavicular block is valuable as it offers another option to the regionalist. The supraclavicular approach represents an effective and non-inferior alternative to the infraclavicular approach with respect to effective analgesia and safety.
